# Real-world effectiveness and persistence of reference etanercept versus biosimilar etanercept GP2015 among rheumatoid arthritis patients: A cohort study

**DOI:** 10.3389/fphar.2022.980832

**Published:** 2022-10-03

**Authors:** Nuria Carballo, Carolina Pérez García, Santiago Grau, Jordi Monfort, Xavier Durán-Jordà, Daniel Echeverría-Esnal, Olivia Ferrández

**Affiliations:** ^1^ Pharmacy Department, Hospital del Mar—Parc de Salut Mar, Barcelona, Spain; ^2^ Universitat Autònoma de Barcelona, Barcelona, Spain; ^3^ Rheumatology Department, Hospital del Mar—Parc de Salut Mar, Barcelona, Spain; ^4^ Methodology and Biostatistics Support Unit, Institute Hospital del Mar for Medical Research (IMIM), Barcelona, Spain

**Keywords:** GP2015, etanercept (enbrel), biosimilar agents, effectivenes, rheumatoid anhritis, drug survival real-life data

## Abstract

Although several randomized clinical trials have confirmed that there is no difference in efficacy between etanercept and its biosimilar versions in the treatment of rheumatoid arthritis (RA), limited real-world evidence is available. We conducted a cohort study to compare the effectiveness and treatment persistence between the reference etanercept (ETN) and the biosimilar GP2015 in RA patients in a real-life setting. Adults with a diagnosis of RA who initiated treatment with ETN or GP2015, between January 2007 and December 2019, were included. The follow-up period was 52 weeks. The primary outcome was the mean of change in the DAS28-CRP values and the adjusted mean difference from baseline to 52 weeks between ETN and GP2015. Other effectiveness endpoints assessed were the rate of patients who achieved remission or low disease activity (LDA) at week 52, who showed a reduction of DAS28-CRP value greater than or equal to 1.2 from baseline to week 52 and rate of good responder patients (those meeting both effectiveness measures) at week 52. Treatment effectiveness over time (baseline, 26 and 52 weeks) was compared between the ETN and GP2015 groups using mixed effects models. Treatment persistence (probability of maintaining the same treatment over time) was also evaluated and shown using Kaplan–Meier survival curves. A total of 115 RA patients were included (ETN, *n* = 90; GP2015, *n* = 25). No differences were observed in the primary outcome: DAS28-CRP score decreased from baseline to week 52 [5.1 to 2.7 (mean of change -2.37) in ETN group and 5.0 to 2.2 (mean of change -2.84) in GP2015 group, *p*-value = 0.372] and the adjusted mean difference was −0.37 (−1.03 to 0.29). No differences were also observed in the other effectiveness endpoints assessed among patients treated with ETN or GP2015: rate of patients who achieved remission (54.1% vs. 66.7%, *p*-value = 0.303) and LDA (71.6% vs. 80.9%, *p*-value = 0.391) at week 52, reduction of DAS28-CRP value greater than or equal to 1.2 from baseline to week 52 (75.6% vs. 80.9%, *p*-value = 0.613) and rate of good responder patients (58.1% vs. 76.1%, *p*-value = 0.202). Drug survival was 82% and 80% for ETN and GP2015, respectively (log-rank *p*-value = 0.804). Etanercept and its biosimilar GP2015 show similar effectiveness and treatment persistence in RA patients in a real-life setting.

## 1 Introduction

Rheumatoid arthritis (RA) is a chronic, autoimmune, and inflammatory disease that can lead to accumulating joint damage and disability. This pathology can cause significant morbidity and decreased quality of life. RA is a heterogeneous disease, with variable clinical presentation and pathogenic mechanisms in which both genetic and environmental factors may be involved (Smolen et al., 2018).

Its prevalence in the adult population ranges around 0.5–1% worldwide (Alamanos et al., 2006), being of approximately 0.5% in Spain (Silva-Fernández et al., 2020). However, there is a considerable variation among the different populations.

New management recommendations, such as early diagnosis and the implementation of prompt initiation of effective therapy in a treat-to-target approach, have considerably improved RA outcomes (Grigor et al., 2004; Smolen et al., 2016). According to the recommendations of the European League against Rheumatism (EULAR; Felson et al., 2011), the current treatment goal is to achieve disease remission or, at least, a low disease activity (Aletaha et al., 2005).

The emergence of biological disease modifying anti-rheumatic drugs (DMARDs), such as tumor necrosis factor alpha inhibitors (TNFi), has revolutionized the treatment of RA. Five TNFi drugs are currently approved for the treatment of RA: Etanercept, infliximab, adalimumab, golimumab, and certolizumab pegol.

Etanercept was the first TNFi to obtain approval for the treatment of RA in 1998 and currently remains a first-line biological DMARD therapy for RA worldwide (Chadwick et al., 2018). Etanercept is a dimeric fusion protein consisting of the extracellular ligand-binding portion of the human 75 kilodalton (p75) TNF receptor linked to the Fc portion of human IgG1. By competitively inhibiting TNF binding to its cell surface receptors, etanercept prevents TNF-mediated inflammatory cellular responses (Burness and Duggan, 2016).

Despite the significant benefits of these biologicals, the high acquisition cost of these drugs places a significant economic burden on healthcare resources (Curtis and Singh, 2011; Putrik et al., 2014). The expiration of patents protection has led to the development of biosimilar drugs, which substantially reduce the economic impact of treatments and improve drug accessibility (Azevedo et al., 2015).

A biosimilar is a biological agent that contains a version of the active substance of an already authorized original biological medicinal product (reference or originator biologic) and whose similarity to the reference medicinal in terms of quality characteristics, biological activity, safety and efficacy has been proved (European Medicines Agency, 2014).

GP2015 (Erelzi^®^) is the second etanercept biosimilar approved for all indications included in the reference product label (Deeks, 2017; Fitton et al., 2018). Bioequivalence between GP2015 and etanercept originator Enbrel^®^ (ETN) was firstly demonstrated in healthy patients (von Richter et al., 20152017). Through the EGALITY study, in which bio similarity between GP2015 and ETN in patients with plaque psoriasis was demonstrated (Griffiths et al., 2017), GP2015 was approved by the Food & Drug Administration (FDA) and the European Medicines Agency (EMA) in 2016 and 2017, respectively. Efficacy equivalence of both drugs in RA patients was later confirmed following the EQUIRA study (Matucci-Cerinic et al., 2018). However, these randomized controlled trials (RCTs) usually have high internal but low external validity (Haugeberg et al., 2021).

The retention rate of treatment with biological therapy, drug survival or the probability of treatment persistence with the same biological drug (i.e. the probability of maintaining the treatment over time) provides an index of overall drug effectiveness, patient satisfaction and treatment compliance (Wolfe, 1995) (21). However, the evidence in terms of treatment persistence generated in RCTs presents many limitations, mainly due to inherent biases associated with its measurement under optimal conditions. Therefore, it may not be translated into the assessment of persistence in real-world settings (Hernandez et al., 2019).

In this context, it is necessary to generate real-world evidence comparing the originator and biosimilar products of etanercept to guide clinicians in daily practice.

The aim of this study was to compare the effectiveness and treatment persistence between ETN and GP2015 in a real-life cohort of RA patients.

## 2 Materials and methods

### 2.1 Participants

Patients with RA aged 18 years and older, who initiated treatment with ETN between January 2007 and December 2019 and with GP2015 between January 2018 and December 2019, were included in this study. Patients ever treated with etanercept that had undergone a wash-up period of at least 24 weeks were also included. Patients with a diagnosis of a rheumatologic disease other than RA, and patients who had previously received treatment with another biosimilar of etanercept different than GP2015 were excluded.

All patients were identified through pharmacy registry and an expert in RA recorded and assessed these data.

### 2.2 Study design and procedures

This was a retrospective real-life observational cohort study carried out in a tertiary university hospital that serves as a reference hospital for the population residing in one of the four health areas into which the Barcelona public health system divides the territory, and which encompasses around 300,000 residents (Informació, 2022). Prevalence of rheumatoid arthritis in Catalonia is estimated in 0.42% (Fina-Aviles et al., 2016). All patients were visited in the Rheumatology Department and diagnosed with RA according to the 2010 American College of Rheumatology/European League Against Rheumatism collaborative initiative RA classification criteria (Aletaha et al., 2010).

The decision to initiate treatment with ETN or GP2015 was made at the physician’s discretion and considering the fact that GP2015 was introduced at the center in 2018. All treatments are provided free of charge to patients and paid for by the publicly financed National Health Care Service.

The follow-up period was 52 weeks. Study ethical approval was obtained from the Clinical Research Ethics Committee of the center (reference number: CEIm 2021/10022) and the Strengthening The Reporting of Observational Studies in Epidemiology (STROBE) statement guidelines were followed (Von Elm et al., 2007).

Data collected were extracted from the computerized medical record system: demographic data (age, gender, ethnicity, body mass index (BMI)); smoking status; comorbid conditions; disease status (disease duration [defined as the time from RA diagnosis by the rheumatologist to the start of etanercept], clinical disease activity at the start of therapy [measured by the Disease Activity Score-28 using C-reactive protein (DAS28-CRP) and using erythrocyte sedimentation rate (DAS28-ESR)], number of swollen and tender joints (SJC and TJC respectively), presence of bone erosions at study baseline, positive findings for rheumatoid factor (RF) and anti-citrullinated protein antibodies (ACPA), serum levels of C-reactive protein (CRP) and erythrocyte sedimentation rate (ESR) at etanercept introduction; medication data (concomitant DMARDs and corticosteroids, prior treatments [both DMARDs and other biologic agents] and number of previous treatments). Adverse events (AE) during etanercept therapy that were the reason for interruption of treatment were reflected.

DAS28-CRP, DAS28-ESR, TJC, and SJC, serum CRP and ESR levels, use of concomitant DMARDs and corticosteroids were assessed at the baseline, at 26 and 52 weeks after the initiation of ETN or GP2015.

All treatment decisions were at the discretion of the treating physician as part of routine clinical practice and clinical evaluations were performed by the same clinician for all patients.

### 2.3 Study outcome

#### 2.3.1 Effectiveness measurement

The primary outcome of the study was the mean of change in the DAS28-CRP values and the adjusted mean difference from baseline to 52 weeks between ETN and GP2015.

Other effectiveness endpoints assessed were the rate of patients who achieved remission (defined as DAS28-CRP ≤ 2.6) or low disease activity (LDA; defined as DAS28-CRP ≤ 3.2) at week 52 and patients who showed a reduction of DAS28-CRP value greater than or equal to 1.2 from baseline to week 52. The rate of patients meeting both effectiveness measures are classified as good responders according to the European EULAR response criteria (Fransen and van Riel, 2009).

The DAS28-CRP was used as the primary effectiveness measure and was calculated as it is a validated instrument to determine disease activity of RA at any given time point during the disease course (Wells et al., 2009).

Treatment effectiveness was also measured through a longitudinal data analysis. The evolution profile of DAS28-CRP, DAS28-ESR, SJC, TJC, serum CRP, and ESR levels and concomitant use of DMARDs and corticosteroids were evaluated at baseline, 26 and 52 weeks after the initiation of ETN or GP2015 and compared between both groups.

#### 2.3.2 Treatment persistence measurement

Overall persistence was determined according to the ISPOR Medication Compliance and Persistence Work Group definition (i.e., “duration of time from initiation to discontinuation of therapy”; Cramer et al., 2008) and was estimated as the duration of time from etanercept therapy initiation to discontinuation during a follow-up period of 52 weeks. Patients who discontinued treatment during the follow-up period were considered non-persistent. The start of follow-up was defined as the date on which the patient first received etanercept, and the end of follow-up was defined as the date of treatment interruption (last day of supply) for those who discontinued or censored at 52 weeks of follow-up. Temporary treatment interruptions (i.e., due to infections, surgery or poor adherence) of less than 12 weeks’ duration were allowed in accordance with other published studies of persistence with TNFi in RA patients where drug-free intervals of 30 days (Yazici et al., 2009), 60 days (Wu et al., 2008) or 90 days (Grijalva et al., 2007) have been used.

Persistence was retrospectively calculated through the electronic pharmacy dispensing record and etanercept survival rates were estimated by the Kaplan-Meier analysis.

Mean time of treatment and reasons for etanercept discontinuation in those patients with treatment interruption (lack of effectiveness, AEs or pregnancy) were recorded and compared between the two groups.

### 2.4 Sample size calculation

Accepting an alpha risk of 0.05 and a beta risk of 0.2 in a two-sided test, a sample size of 87 subjects in the reference group and 29 in the biosimilar group was deemed necessary to recognize as statistically significant a difference greater than or equal to 1 unit in DAS28-CRP score. The SD is assumed to be 1.66. A patient allocation ratio of 3:1 was anticipated considering the low number of patients that received GP2015 over the study period.

### 2.5 Statistical analysis

Categorical variables were presented as frequencies (number and percentage) and were compared using Chi squared and Fisher exact tests, as appropriate. Continuous variables were described as the mean and standard deviation (SD) and were analyzed using Mann-Whitney U test. In addition, for parameters assessed at baseline, 26 and 52 weeks, differences between groups in each phase were tested separately using Mann-Whitney U test. The non-parametric choice was due because most of the continuous variables did not fulfill the normality assumption and considering the low number of patients in the smaller group.

Effectiveness evaluation was performed through longitudinal data analysis using linear mixed models, introducing a time-per-arm interaction term to test the differences between both groups. Main effect, drug and time, and their interaction were variables taken into account in the model. These analyses were adjusted for potential confounders including baseline imbalances (ethnicity, erosive disease, concomitant corticosteroids) and use of concomitant DMARDs using bivariate screening/stepwise selection. These variables were introduced based on clinical criteria and taking into account their statistical significance in the bivariate analysis.

Drug survival analysis comparing ETN and GP2015 groups was shown using Kaplan–Meier survival curves. Differences between drugs were tested using the log-rank test. The rate of treatment persistence at 52 weeks was also derived from these Kaplan–Meier survival curves and its confidence intervals (CI) were based on beta product confidence procedure (BPCP).

Results were considered as statistically significant at *p*-value<0.05. In addition, *p*-values for baseline characteristics were adjusted for Benjamini and Hochberg correction. Statistical analyses were performed using STATA 15.1 and bpcp package in R.

## 3 Results

**FIGURE 1:**
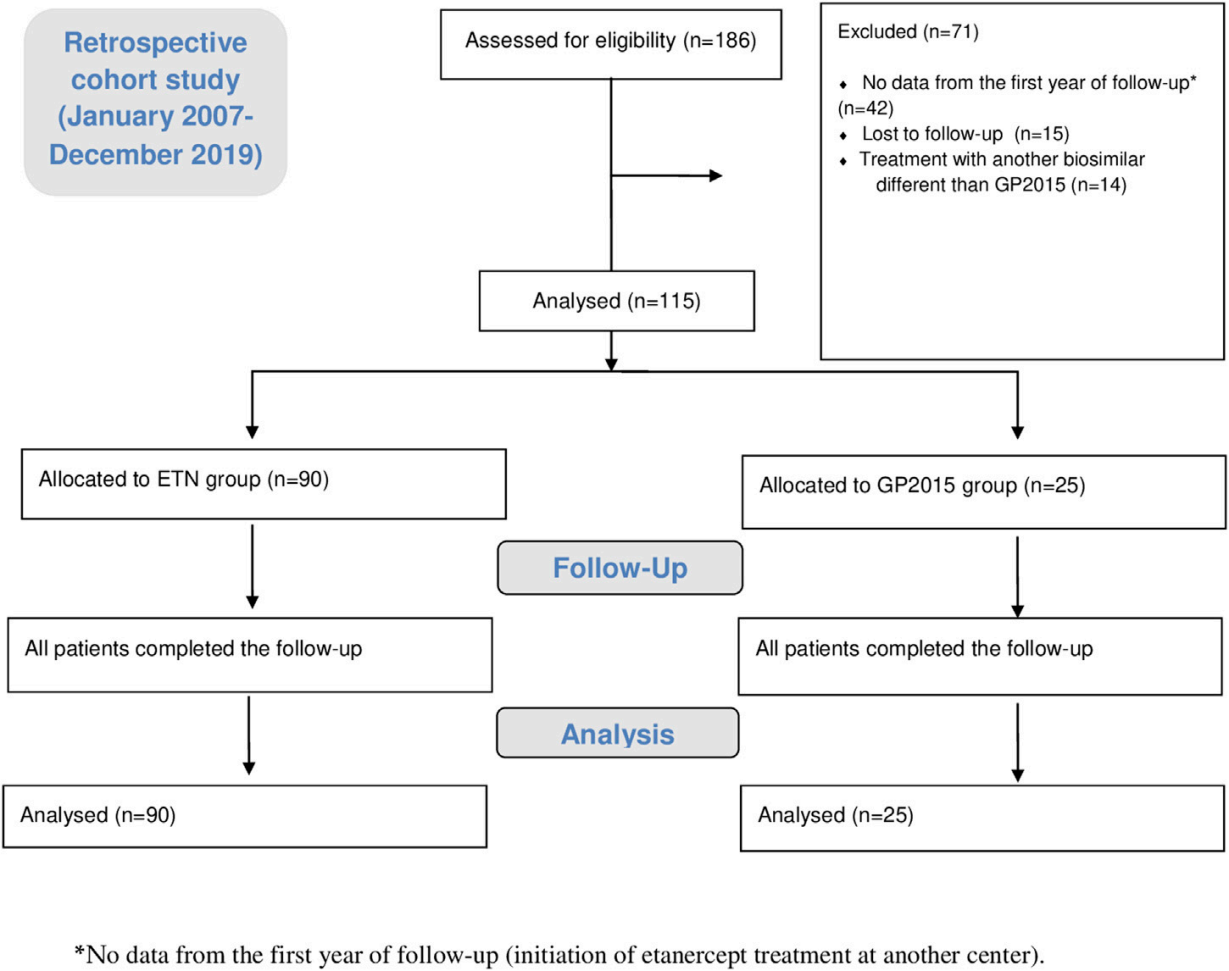
The study flow chart in line with the STROBE: 186 patients were initially assessed for eligibility, of which 71 were excluded. Finally, 115 patients were analyzed, 90 in the group ETN-treated patients and 25 in the group GP2015-treated patients. All patients completed the follow up. STROBE: (Strengthening the Reporting of Observational Studies in Epidemiology).

A total of 186 RA patients initiated etanercept treatment during the study period, of whom 184 were treated for the first time with etanercept and only two had been treated previously. Seventy-one patients were excluded (38.1%) as absence of data from the first year of treatment (42, 22.6%), loss of follow-up (15, 8.1%) and treatment with another biosimilar different than GP2015 (14, 7.5%). Finally, 115 (61.9%) patients were included in the study, 90 patients in treatment with ETN and 25 patients in treatment with GP2015. Figure 1 summarized the study flow chart.

All patients started etanercept therapy at a once-weekly 50 mg subcutaneous injection. Clinical and demographic characteristics of the study population are summarized in Table 1. There were no clinically relevant differences in baseline and demographics characteristics between ETN and GP2015 groups, apart from differences in the ethnicity, a higher proportion of patients with erosive disease, and treatment with concomitant corticosteroids at baseline.

**TABLE 1:** Baseline demographic and clinical characteristics of RA patients treated with etanercept originator or its biosimilar GP2015.

	ETN (*n* = 90)	GP2015 (*n* = 25)	Total (*n* = 115)	p-value	p-value^a^
Demographics					
Age (years) at ETN introduction	53.1 (12.4)	55.9 (10.5)	53.7 (12.1)	0.362	0.766
Female	66 (73.3)	23 (92.0)	89 (77.4)	0.059	0.341
Weight (kg)	68.3 (14.8)	67.9 (17.5)	68.2 (15.3)	0.646	0.865
BMI (kg/m^2^)	25.5 (4.7)	26.4 (6.3)	25.7 (5.1)	0.793	0.920
Ethnicity				**0.002**	**0.017**
Caucasian	78 (86.7)	16 (64.0)	94 (81.7)		
Hispanic (American Indian or Alaska Native)	12 (13.3)	5 (20.0)	17 (14.8)		
Asian	0 (0.0)	3 (12.0)	3 (2.6)		
African American	0 (0.0)	1 (4.0)	1 (0.9)		
Smoking history			
Current smoker	13 (15.1)	4 (16.0)	17 (15.3)	1.000	1.000
Ex-smoker	14 (16.3)	5 (20.0)	19 (17.1)	0.763	0.920
Comorbidities					
Cardiovascular disease	26 (28.9)	11 (44.0)	37 (32.2)	0.255	0.564
Osteoporosis	31 (34.4)	7 (28.0)	38 (33.0)	0.635	0.865
Lung disease	14 (15.6)	3 (12.0)	17 (14.8)	1.000	1.000
Depression/anxiety	21 (23.3)	9 (36.0)	30 (26.1)	0.209	0.564
Diabetes	6 (6.7)	3 (12.0)	9 (7.8)	0.406	0.773
Glaucoma	3 (3.3)	3 (12.0)	6 (5.2)	0.116	0.443
Thyroid disorder	18 (20.0)	10 (40.0)	28 (24.3)	0.063	0.341
Disease status					
Duration of RA (years)	9.4 (6.9)	8.2 (7.1)	9.2 (6.9)	0.416	0.773
DAS28-CRP	5.1 (1.5)	5.0 (1.1)	5.1 (1.4)	0.655	0.865
DAS2-ESR	5.3 (1.6)	5.3 (1.2)	5.3 (1.5)	0.673	0.865
Erosive disease	79 (91.9)	16 (64.0)	95 (85.6)	**0.002**	**0.017**
RF positive	63 (73.3)	20 (80.0)	83 (74.8)	0.606	0.865
ACPA positive	57 (66.3)	21 (84.0)	78 (70.3)	0.135	0.443
CRP serum levels (mg/dl)	1.7 (2.5)	0.8 (0.7)	1.5 (2.2)	0.236	0.564
ESR serum levels (mm/h)	26.7 (20.6)	25.4 (21.6)	26.4 (20.7)	0.626	0.865
Prior therapy					
Number of previous DMARDs	2.2 (1.0)	1.9 (1.0)	2.2 (1.0)	0.154	0.443
Previous DMARDs				0.199	0.865
1	24 (26.7)	10 (40)	34 (29.6)		
2	33 (36.7)	10 (40)	43 (37.4)		
3	24 (26.7)	2 (8)	26 (22.6)		
≥4	9 (10.0)	3 (12.0)	12 (10.4)		
Number of previous biologic agents	0.3 (0.6)	0.2 (0.5)	0.3 (0.6)	0.430	0.654
Biologic-naïve	66 (73.3)	21 (84.0)	87 (75.7)	0.429	0.773
Previous biologic agents				0.627	0.564
1	20 (22.2)	3 (12.0)	23 (20.0)		
2	3 (3.3)	1 (4.0)	4 (3.5)		
3	1 (1.1)	0 (0.0)	1 (0.9)		
Concomitant therapy			
Concomitant DMARDs			
Yes	69 (78.4)	19 (76.0)	88 (77.9)	0.789	0.920
MTX	37 (41.1)	9 (36.0)	46 (40.0)	0.818	0.920
MTX dose (mg/week)	19.29 (4.8)	20 (6.1)	19.43 (5.1)	0.503	0.835
Leflunomide	21 (23.3)	10 (40)	31 (27.0)	0.126	0.443
Leflunomide dose (mg/day)	17.6 (4.4)	19 (3.2)	18.1 (4.0)	0.540	0.865
Other	12 (13.3)	0 (0.0)	12 (10.4)	0.066	0.341
Concomitant corticosteroids					
Yes	65 (72.2)	7 (28.0)	72 (62.6)	**<0.001**	**0.004**
Prednisone equivalent dose					
≥10 mg/day	35 (55.6)	4 (57.1)	39 (55.7)	1.000	1.000
<10 mg/day	28 (44.4)	3 (42.9)	31 (44.3)		

a Benjamini & Hochberg correction. Data presented as mean (SD) and n (%). ETN etanercept originator (Enbrel^®^), GP2015 etanercept biosimilar (Erelzi^®^), RA rheumatoid arthritis, BMI body mass index, DAS28-CRP disease activity score 28 using c-reactive protein, DAS28-ESR disease activity score 28 using erythrocyte sedimentation rate, RF rheumatoid factor, ACPA anti-citrullinated protein antibody; CRP c-reactive protein, ESR erythrocyte sedimentation rate, MTX methotrexate, DMARDs disease modifying antirheumatic drugs.

Statistically significant.

### 3.1 Effectiveness measurement

The DAS28-CRP score for the ETN-treated patients decreased significantly from 5.1 (1.5) at baseline to 2.7 (1.2) at 52 weeks; the mean (SD) of change was −2.37 (1.66). The GP2015-treated patients also significantly improved DAS28-CRP from 5.0 (1.1) at baseline to 2.2 (1.3) at 52 weeks; the mean (SD) of change was −2.84 (1.91). There was no statistically significant difference (*p*-value = 0.372) between both groups. The adjusted mean difference for DAS28-CRP at week 52 between ETN and GP2015 was −0.37 (95% CI: −1.03 to 0.29).

**FIGURE 2:**
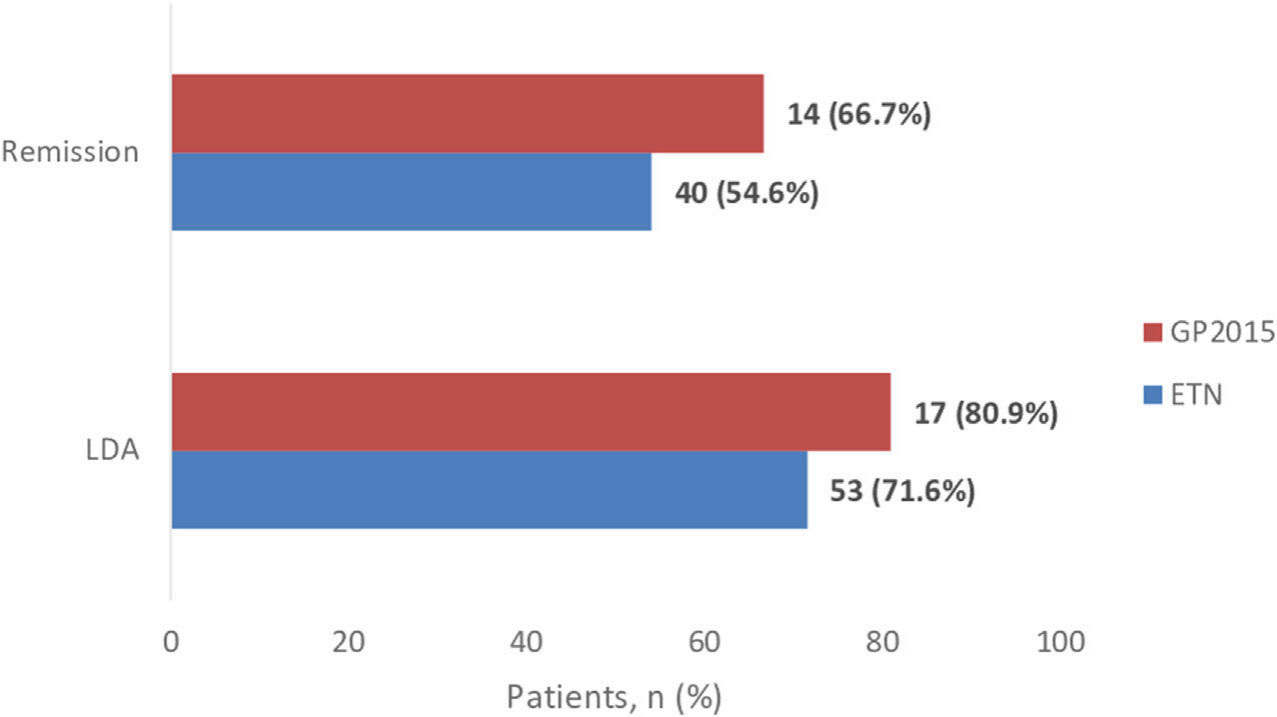
Proportion of patients achieving remission defined as DAS28-CRP ≤2.6 and low-disease activity score (LDAS) defined as DASR28-CRP ≤3.2. The rate of patients who achieved remission at week 52 were 40 (54.6%) and 14 (66.7%), *p*-value = 0.303, in the ETN and GP2015 group, respectively. Fifty-three (71.6%) patients in the ETN group and 17 (80.9%) in the GP2015 group (*p* value = 0.391) achieved LDAS.

**FIGURE 3:**
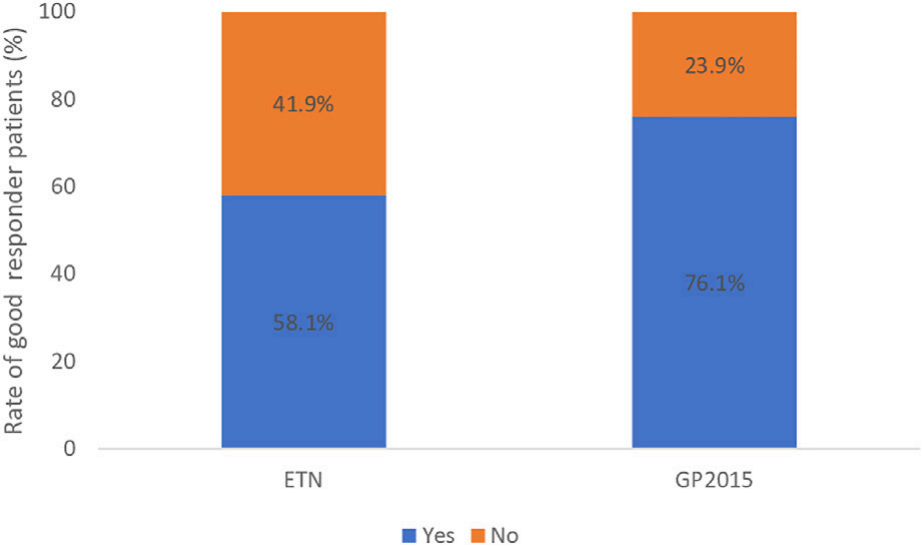
Rate of good responder patients: who achieved low disease activity (DAS28-CRP ≤ 3.2) at week 52 and show a significant change of 1.2 in DAS28-CRP from baseline to 52 weeks. It was reached in 43 (58.1%) and 16 (76.1%) patients in the ETN and GP2015 group respectively, *p*-value = 0.202.

The rate of patients who achieved remission and LDA at week 52 and the rate of patients with good response are shown in Figure 2 and Figure 3, respectively.

Fifty-six (75.7%) patients and 17 (80.9%) in the ETN and GP2015 group, respectively showed a reduction of DAS28-CRP value greater than or equal to 1.2 from baseline to week 52, *p*-value = 0.613.

The evolution profile of DAS28-CRP and DAS28-ESR are presented in Table 2 and evolution profile of SJC, TJC, serum CRP and ESR levels and concomitant use of DMARDs and corticosteroids are presented as additional files.

**TABLE 2:** Evolution of DAS28 at baseline, at 26 and 52 weeks after the initiation and comparison between ETN and GP2015 groups.

	ETN (*n* = 90)	GP2015 (*n* = 25)	Total (*n* = 115)	*p*-value
DAS28-CRP				
Baseline	5.1 (1.5)	5.0 (1.1)	5.1 (1.4)	0.897
At 26 weeks	2.6 (1.1)	2.3 (1.5)	2.6 (1.2)	0.243
At 52 weeks	2.7 (1.2)	2.2 (1.3)	2.6 (1.2)	0.103
DAS28-ESR				
Baseline	5.3 (1.6)	5.3 (1.2)	5.3 (1.5)	0.425
At 26 weeks	2.6 (1.2)	2.3 (1.7)	2.5 (1.3)	0.992
At 52 weeks	2.6 (1.3)	2.1 (1.5)	2.5 (1.3)	0.377

Data presented as mean (SD) and n (%). ETN etanercept originator (Enbrel^®^), GP2015 etanercept biosimilar (Erelzi^®^), DAS28-CRP disease activity score 28 using c-reactive protein, DAS28-ESR disease activity score 28 using erythrocyte sedimentation rate. The differences observed in the baseline *p*-values compared to Table 1 are derived from the longitudinal statistical analysis used.

**FIGURE 4:**
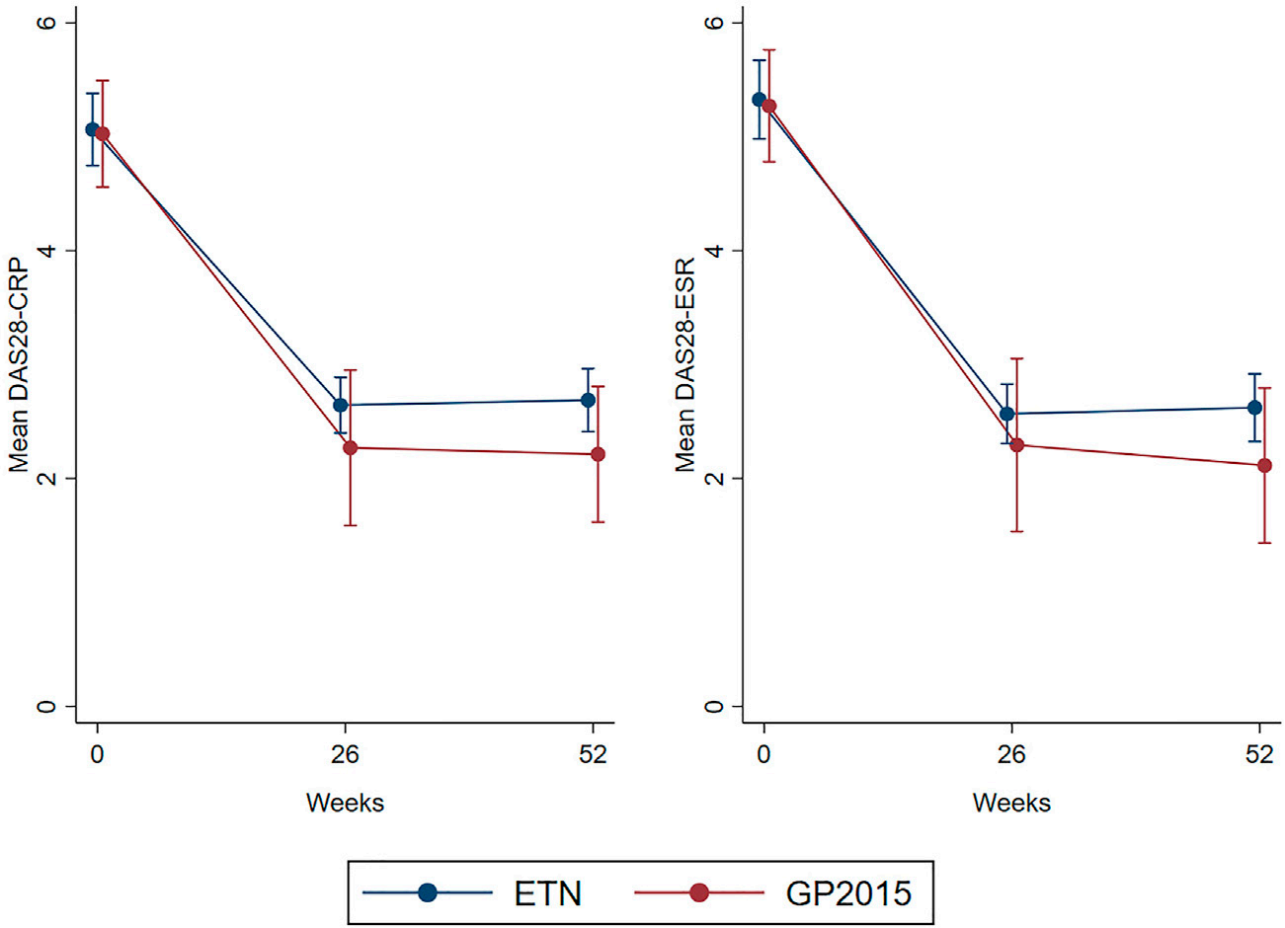
Mean change from baseline over 52 weeks in DAS28-CRP and DAS28-ESR scores of ETN and GP2015 groups. DAS28-CRP disease activity score 28 using c-reactive protein, DAS28-ESR disease activity score 28 using erythrocyte sedimentation rate, ETN etanercept originator (Enbrel^®^), GP2015 etanercept biosimilar (Erelzi^®^). DAS28-CRP (*p*-value = 0.263). DAS28-ESR (*p*-value = 0.293)

Figure 4 shows the changes over time in DAS28-CRP and DAS28-VSG values and the comparison between ETN and GP2015 during the 52-weeks study period, without statistical differences. There were also no differences in the time course of SJC, TJC, serum CRP and ESR levels (figures provided as additional data).

### 3.2 Treatment persistence measurement

**FIGURE 5:**
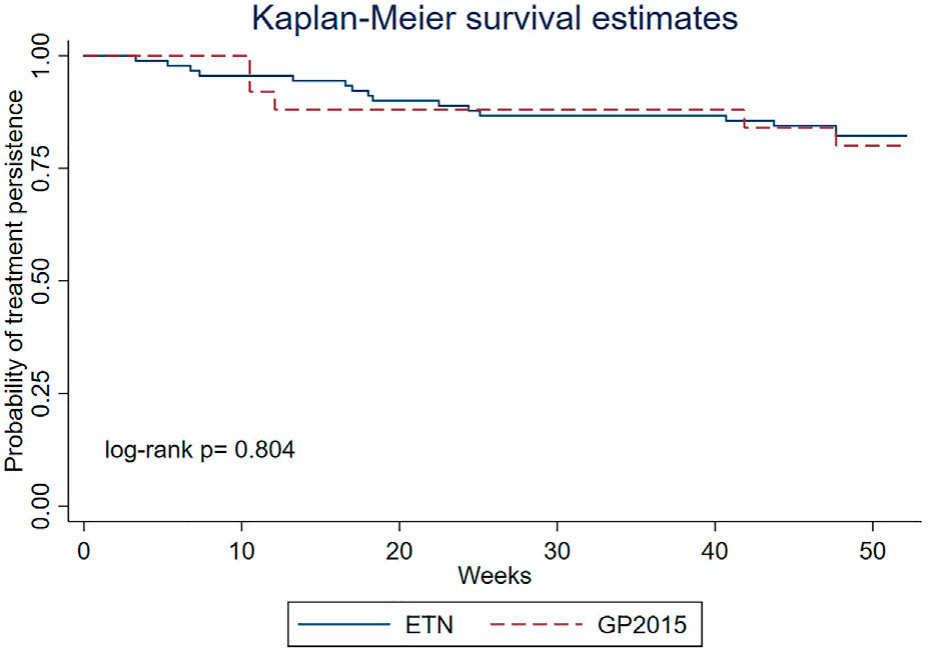
Overall treatment persistence with ETN and GP2015 in RA patients shown as the fraction (between 1 and 0) of patients remaining on therapy during 52 weeks after therapy initiation (long-rank *p* = 0.804).

Drug survival rates estimated by the Kaplan-Meier analysis are shown in Figure 5. The rate of treatment persistence at 52 weeks was 0.82 (95% CI: 0.72–0.89) for ETN and 0.80 (95% CI: 0.59–0.93) for GP 2015.

Mean (SD) time of treatment was 46.74 (12.97) weeks and 46.51 (13.54) weeks for ETN and GP2015, respectively (*p*-value = 0.686). In non-persistence patients (patients who discontinued treatment during follow-up) this value was 22.35 (15.03) and 24.53 (18.60) weeks, respectively (*p*-value = 1.000).

Reasons for etanercept discontinuation comparing the ETN and GP2015 groups were lack of effectiveness in 7 (7.8%) patients and in 4 (16%) patients, AEs in 7 (7.8%) and in 1 (4%) patient, and pregnancy in 2 (2.2%) and in 0 (0.0%) patients, respectively. No statistical differences between the two groups (*p*-value = 0.301) were observed. Reported AEs are described in a table as additional. No other patients experienced any AE during follow-up period.

## 4 Discussion

In the present real-world analysis of RA patients treated with etanercept, no differences in effectiveness and treatment persistence for ETN compared to its biosimilar GP2015 during the first year of treatment were observed.

Baseline demographics and clinical characteristics were similar in both groups. However, differences in ethnicity were observed, with significantly more Caucasian patients in the ETN group. This difference may be explained by the recent increase in the number of non-Caucasian population in Spanish society (related to the increase in migratory flows in recent years (Ministerio de Inclusión Seguridad Social y migraciones, 2020)) together with the fact that ETN was marketed almost 20 years before GP2015. A significantly higher proportion of patients with erosive disease and in treatment with corticosteroids therapy at baseline were also observed in the ETN group. Bone erosions are generally associated with disease severity and consistent with advanced disease (Fuchs et al., 1989) and these findings could be justified by the update in RA treatment strategies, which lately employ early initiation of DMARDs and concomitant short-term corticosteroids to suppress inflammation, especially in early RA (Crossfield et al., 2021; Smolen et al., 2020).

The results of the present study are consistent with those of clinical trials, as the EQUIRA study, in which efficacy equivalence between GP2015 and ETN in patients with RA was demonstrated at 24 weeks (Matucci-Cerinic et al., 2018). In this study the mean (SD) DAS28-CRP change from baseline to week 24 was similar between the GP2015 [−2.78 (1.1)] and ETN groups [−2.78 (1.0)]. Efficacy was assessed using the DAS28-CRP score, in the same way as we proceeded in our study.

Treatment effectiveness has generally been determined by comparing group means of changes in disease activity variables. However, a significant difference between groups does not reflect the number of patients who responded to treatment (Fransen and van Riel, 2009). The present study therefore assessed the rate of patients who achieved remission or LDA and showed a reduction of DAS28-CRP value greater than or equal to 1.2 from baseline to week 52 and confirmed that no differences were found between both groups (*p*-value = 0.202). Although no statistically significant differences were observed, we are aware that 58.1% of ETN-patients versus 76.1% of GP2015-patients achieved LDA and showed a significant reduction in DAS28-CRP at 52 weeks (≥1.2 from baseline). This could be in line with previously mentioned about updates in treatment strategy in recent years with a possible prompt therapy initiation in GP2015 patients.

A fundamental limitation of clinical trials is that the included patients generally differ from those attended in daily practice, and patients in a normal clinical setting would be ineligible for such trials (Ugalde et al., 2021). This fact could account for some differences between the EQUIRA and the present study, such a shorter evaluation period (24 weeks in front of 52 weeks in the present study) and inclusion of patients with active disease in the first one. Although RCTs are considered the gold standard for assessing efficacy, their results are often limited because of their rigorous inclusion criteria. Furthermore, the generalizability of their findings is often limited because of the lack of extrapolation of real-life setting (Zink et al., 2006; Aaltonen et al., 2017).

Considering previous published real-life data, Atzeni et al. (2021) evaluated in a retrospective double center study effectiveness and safety of biosimilar SB4 (Benepali^®^) and ETN in RA real-life patients. At 6 months the DAS28-CRP score was not different between the 11 patients receiving first-line biosimilar etanercept and 51 patients receiving the originator (2.3 ± 1.2 versus 2.7 ± 1.3; *p* > 0.05). Other scores as DAS28-ESR, Clinical Disease Activity Index (CDAI) and Simple Disease Activity Index (SDAI) were evaluated, without significant differences.

Codreanu et al. also demonstrated similar findings. Similar effectiveness and safety were shown between biosimilar SB4 and ETN after the first 6 months of treatment in a real-life national cohort of RA patients. After this period the DAS28-CRP score was 3.3 ± 1.3 in both 123 patients treated with etanercept and 119 patients treated with SB4 with a *p* = 0.829 (Codreanu et al., 2019). DAS28 remission and Boolean remission were achieved by 18.7% in ETN group and 17.6% in SB4 group (*p* = 0.823), and by 11.4% in ETN group and 11.8% in SB4 group (*p* = 0.926), respectively. Similar to the study of Atzeni et al., DAS28-ESR, CDAI and SDAI scores were also evaluated without significant differences.

Both real-life studies evaluated a 6 months-treatment period. However, in our study a period of 12 months was considered, since assessments of disease activity should be made at 12 months after start of treatment in maintenance trials (Committee for Medicinal Products for Human Use, 2017). Furthermore, both studies compare the effectiveness and safety of etanercept originator and its biosimilar SB4 and our study compares effectiveness with a different biosimilar product, GP2015. To our knowledge, this is the first real-world study evaluating effectiveness between ETN and GP2015, as all other published studies evaluated the effectiveness of other etanercept biosimilar products, mainly SB4.

Real-world treatment persistence has been considered a proxy measure not only for treatment safety and effectiveness, but also for patient satisfaction (Luttropp et al., 2019). Evaluation of survival times and discontinuation rates associated with treatment is necessary when evaluating their real-world effectiveness, however there are many reasons associated with treatment modification in addition to lack of effectiveness such as AE, pregnancy, or death. RCTs are relatively short compared with the chronic course of RA and therefore real-world studies with longer patient follow-up can be very useful.

In the present study, survival probability curves were highly similar for the ETN and GP2015. Although previous studies comparing TNFi persistence in patients with RA reported conflicting results, our persistence rates at 52 weeks (82% for ETN and 80% for GP2015) appear to be higher than those reported in other publications, considering the inclusion of both biological- naïve and non-naïve patients. A recent systematic literature review and meta-analysis reported 12-months survival rates in the first-line and second-line treatment setting for etanercept originator of 71% and 61%, respectively (Emery et al., 2020). In other study involving 6,153 biological-naïve RA patients, persistence rates of 82% for etanercept up 52 weeks were reported (Sruamsiri et al., 2018). Among non-persistent patients, there was no difference in mean treatment duration.

As we wanted to show the global persistence, all reasons for treatment discontinuation have been evaluated. The most prevalent reasons for discontinuation are lack of effectiveness and AEs in both groups. Although no statistically significant differences were observed in the reasons for drug discontinuation, discontinuation due to a lack of effectiveness was higher in patients treated with the biosimilar and it may be due to an earlier switch of therapy comparing to previous years due to an increase in the currently available biological drugs alternatives (Tak and Kalden, 2011; Conti et al., 2013).

The study carried out presents several limitations, mainly related to its retrospective nature, which may lead to missing or incomplete data. It consists of a non-randomized single-center observational study. The prescription bias could be present, although no significant differences were found at baseline characteristics in terms of disease activity. A limitation which stands out above the rest is the difference in sample size between the study groups, with a particularly small sample size for the GP2015 group. Moreover, 25 patients were included out of the 29 expected for the GP2015 group which represented a loss of 13,8% with respect the calculated sample size. It was limited by the number of RA patients treated in a tertiary hospital, the rise of variety of treating RA drugs other than etanercept in recent years, and the fact that at the time the study was initiated, GP2015 had just been marketed. Another limitation is that some baseline differences are observed between the two study populations because of the difference in time between both treatment strategies; however, this is a common occurrence in retrospective analyses. Pain and fatigue assessments, key patient data, were not available also due to the retrospective nature of the study. Finally, we acknowledge that a sensitivity analysis with all included patients would have been of interest. However, we have waived such analysis for the following reasons: the lack of primary outcome measure in patients with loss to follow-up and the use of other biosimilars, which would have increased heterogeneity.

The strengths of our study are based on data collection from a real-life clinical setting and a longer follow-up time on the measure of effectiveness than other real-life studies. Another strength of the study is the scarcity of missing data which contributes to reduce selection bias and increase statistical power.

To the authors’ knowledge, no previous analysis comparing drug survival between etanercept originator and its biosimilar GP2015 in patients with RA in real-life setting had been carried out, so these findings are the first reported in this field.

In summary, ETN and its biosimilar GP2015 showed similar effectiveness and treatment persistence in RA patients in a real-life setting. These results should be confirmed in other observational studies with larger sample sizes.

## Data Availability

The original contributions presented in the study are included in the article/Supplementary Material, further inquiries can be directed to the corresponding authors.
