# Supplementary material for: Real-world effectiveness and persistence of reference etanercept versus biosimilar etanercept GP2015 among rheumatoid arthritis patients: A cohort study
Source: Front Pharmacol. 2022 Oct 3;13:980832. doi: 10.3389/fphar.2022.980832 (PMC9575986; doi:10.3389/fphar.2022.980832)
Supplement: Supplementary file 1 [file Presentation1.pdf]

Supplementary Materials

Table S1. Evolution profile of TJC, SJC, serum CRP and ESR levels and concomitant use of DMARDs and corticosteroids at baseline, at 26 and 52 weeks after the initiation and comparison between ETN and GP2015 groups.

	ETN (n=90)	GP2015 (n=25)	Total (n=115)	p-value
**TJC**				
Baseline	9.0 (7.9)	8.6 (6.6)	8.9 (7.6)	0.711
At 26 weeks	1.2 (3.0)	1.8 (4.1)	1.3 (3.3)	0.561
At 52 weeks	1.6 (3.2)	1.4 (2.3)	1.6 (2.9)	0.824
**SJC**				
Baseline	8.9 (7.4)	6.8 (5.0)	8.4 (7.0)	**0.037**
At 26 weeks	1.4 (2.7)	1.8 (2.9)	1.5 (2.7)	0.662
At 52 weeks	1.5 (2.8)	1.4 (2.1)	1.5 (2.7)	0.892
**CRP serum levels (mg/dL)**				
Baseline	1.7 (2.5)	0.8 (0.7)	1.5 (2.2)	**0.011**
At 26 weeks	0.8 (1.6)	0.4 (0.6)	0.7 (1.5)	0.408

At 52 weeks	0.5 (0.8)	0.3 (0.5)	0.5 (0.7)	0.568
**ESR serum levels (mm/h)**				
Baseline	26.7 (20.6)	25.4 (21.6)	26.4 (20.7)	0.728
At 26 weeks	18.7 (17.2)	14.0 (14.2)	17.7 (16.7)	0.236
At 52 weeks	18.4 (16.4)	11.8 (15.6)	16.9 (16.3)	0.089
**Concomitant DMARDs**				
Baseline	69 (78.4)	19 (76.0)	88 (77.9)	0.798
At 26 weeks	56 (70.9)	13 (59.1)	69 (68.3)	0.544
At 52 weeks	50 (69.4)	13 (59.1)	63 (67.0)	0.605
**Concomitant corticosteroids**				
Baseline	65 (72.2)	7 (28.0)	72 (62.6)	**<0.001**
At 26 weeks	45 (55.6)	6 (27.3)	51 (49.5)	**0.012**
At 52 weeks	36 (48.6)	4 (18.2)	40 (41.7)	**0.009**

Data presented as mean (SD) and n (%). ETN etanercept original (Enbrel®), GP2015 etanercept biosimilar (Erelzi®), TJC tender joint count, SCJ swollen joint count, CRP c-reactive protein, ESR erythrocyte sedimentation rate, DMARDs disease

modifying antirheumatic drugs. The differences observed in the baseline p-values compared to Table 1 are derived from the longitudinal statistical analysis used.

Table S2. Reported adverse events leading to discontinuation in the ETN and GP2015 groups.

Adverse events	ETN, n=90	GP2015, n=25
Total	7 (7.8)	1 (4.0)
Injection site reactions	1 (1.1)	0 (0.0)
Upper respiratory tract infection	1 (1.1)	0 (0.0)
Urticaria	2 (2.2)	0 (0.0)
Hepatotoxicity	1 (1.1)	0 (0.0)
Thrombocytopenia	1 (1.1)	0 (0.0)
Pneumonia	1 (1.1)	0 (0.0)
Flu-like syndrome	0 (0.0)	1 (4.0)

Data presented as n (%). ETN etanercept original (Enbrel®), GP2015 etanercept biosimilar (Erelzi®)

Figure S1. Mean change from baseline over 52 weeks in **a)** TJC **b)** SJC **c)** CRP **d)** ESR scores of ETN and GP2015 cohorts.

ETN etanercept original (Enbrel®), GP2015 etanercept biosimilar (Erelzi®), TJC number of tender joints, SJC number of swollen joints, CRP C-reactive protein, ESR erythrocyte sedimentation rate,

a) TJC (p-value=0.679)

b) SJC (p-value=0.209)

c) CRP (p-value=0.190)

d) ESR (p-value=0.251)
