# Supplementary figures and images for: Real-world effectiveness and persistence of reference etanercept versus biosimilar etanercept GP2015 among rheumatoid arthritis patients: A cohort study
Source: Front Pharmacol. 2022 Oct 3;13:980832. doi: 10.3389/fphar.2022.980832 (PMC9575986; doi:10.3389/fphar.2022.980832)

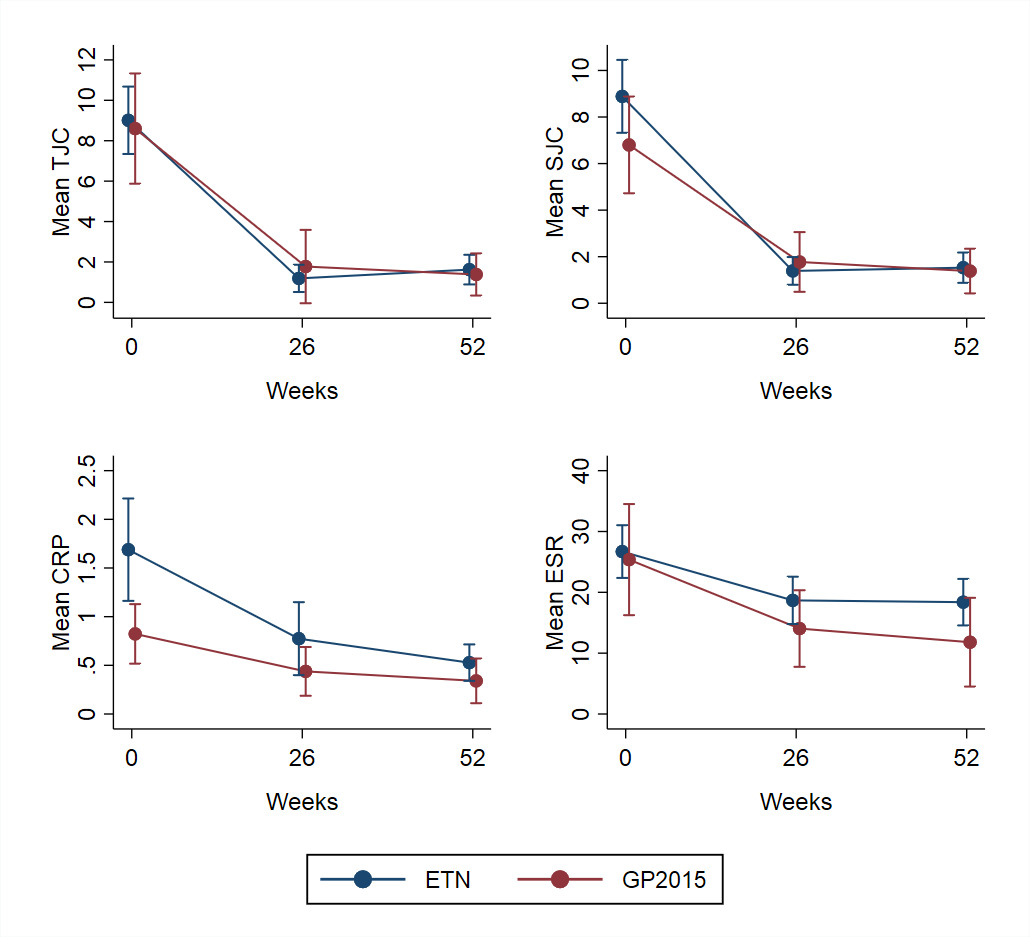

Supplement: Supplementary file 2 [file Image1.jpg]
